# The Scandal of Out-Patients

**Published:** 1911-10-07

**Authors:** 


### THE SCANDAL OF OUT-PATIENTS.

### Wake Up Somebody!

In our voluntary hospitals the managers and the administrative officials make a point of visiting and inspecting the wards; the patients' friends visit the institutions weekly; in short, the full glare of publicity—that publicity which has done so much for our voluntary institutions—falls upon the in-patient arrangements with commendably advantageous results to the welfare of every sick patient who is fortunate enough to be admitted to a ward.

The case, unfortunately, is widely different when we come to many out-patient departments. In the ill-lighted, badly-ventilated, horribly overcrowded little rooms that still serve to accommodate hundreds of patients who are waiting their turn, few take an active interest. The out-patient department is neglected, and it gains next to nothing from that informative and constructive criticism which has been so pregnant of good results in the case of the in-patient department.

What is the condition of some out-patient departments in, say, a large London hospital? We have no isolated case in mind when we sketch this condition: the résumé here given applies, unfortunately, to many hospitals, some of which, still more unfortunately, have not so very long ago spent comparatively large sums in improving and enlarging the out-patient department. Let us imagine the reader enters the department at midday. Whether in summer or winter makes no difference, he will find the waiting-room, if there is one, uncomfortably, distressingly hot, not merely through the actual raising of the temperature by natural or artificial heating, but by the unpleasant, moist, "oily" heat which emanates from a large body of people whose bodies are not in that state of cleanliness which is only possible to those who are as solicitously careful of their skins as Johnson said he was of his belly. There is no allegation here that out-patients, as a class, are unclean; but they give out, when cooped up in a hot and close room, flurried, as most of them are, by hurry, worry, and anxiety, that characteristically human effluvium which, when it is detected in a hall or building, at once raises the justifiable suspicion that the place is badly ventilated.

He will notice, further, that there is no effective system of isolation. The clean patient cannot, without trouble, and sometimes much trouble, safeguard himself from contact with the indifferently clean: the mother bringing her little girl to be operated on for adenoids must seat herself next to the chronic with varicose veins, with the result that during the waiting time the little girl hears a number of doubtless interesting but certainly unnecessary comments upon various diseases, their prognosis and complications. One by one the waiters drop into the examining rooms; one by one they pass out again, in those departments which have no separate exit for the "already seens," to mingle with the new, and undergo yet a further trial of patience, endurance, good temper, and resignation, by waiting their turn in a long queue which slowly moves past the dispenser's wicket.

The mere walk through the department will convince him that the indictment brought against it is not merely academic. The counts in it are deserving of careful attention. They are overcrowding, bad air consequent upon overcrowding and imperfect ventilation and bad heating, a want of system and organisation which results in inflicting numerous unnecessary trials, indignities, and dangers upon the patients.

It is with a full sense of our responsibility that we publish this indictment. It is with a full confidence in the energy of the managers of our voluntary institutions that we say these defects need only to be known to be condemned, swept away, and replaced by a better and more enlightened system of dealing with the out-patient.

Take the position for a moment from the point of view of the average patient—that of the man or woman who comes to the hospital not with the intention of abusing but of using the out-patient department. Such a patient is not gravely ill in the sense that he or she needs in-treatment urgently; the disease for which patients come to *Outers* are usually chronic, not acute ones, for the acute cases have already been eliminated and dealt with in the front surgery, casualty department, or admission block. But it is just the chronic sufferer who is liable to attacks of acute infectious disease, using the term in its widest sense and not limiting it to those cases of illness which are now, very properly, promptly isolated. The "bronchitis and emphysema," the "varicose ulcer" attending for his weekly support of Unna's paste bandages, the "anæmia and general debility," the doubtfully compensated heart case—all these have their powers of resistance to the invasion of microbic disease considerably lowered even in the best environment. How much more powerfully must that infective influence act upon them in atmospheres so polluted with germs, so foul with emanations, so fœtid with *Menschenduft* as are the out-patient waiting-rooms in most hospitals in this country? We are not alarmists, we have no desire to overstate the case, but it is obvious that the first impression the average patient obtains when he enters the out-patient department is one of disgust tempered by anxiety. That is to say, if he is one who has the slightest appreciation of the benefits of pure air, and an acquaintance with the discomforts and dangers which follow even a temporary sojourn in such a vitiated atmosphere.

This is what Dr. "Stephen Andrew" wrote in The Hospital, June 17, 1911 ("Hospitals from the point of view of the G.P."):—

It is amazing that patients put up with the treatment they receive in the out-patient department of many hospitals. To begin with, they have to arrive at the hospital at some fixed hour, which is often uncomfortably early. This may be a small matter to those whose homes are close to the hospital, but it is anything but a small matter to those who come from a distance. It means, not infrequently, that the unfortunate mother of a sick child is forced to hurry off with her invalid to catch an early train, leaving the rest of her children to get off to school on their own account—often without any breakfast. Then, arrival by the fixed time does not by any means result in prompt attention and speedy release. On the contrary, the average out-patient may count upon a wait of several hours in an uncomfortable, evil-smelling, draughty waiting-hall. He will be one of a great mass of closely-packed persons, many of whom will be dirty—some offensively so; many of whom will be suffering from diseases which are openly repulsive—such as lupus, impetigo, or some other skin affection; some of whom may be vigorously infectious. The out-patient must take his chance. Luck may give him for neighbours several clean, pleasant folk, or it may provide him with a man with scabies on his right, a woman with pediculi on his left, a child with scarlet fever in front, and a youth in an advanced stage of consumption behind.

From first to last it is no unusual thing for a visit to the out-patient department of a big hospital to consume the greater part of a working day, and that, to a working-man, or a working-man's wife, is an extremely serious matter. In the case of the man it means that the advice and medicine he has received—gratis and from a voluntary charity, be it remembered—has cost him a sixth part of his week's earnings. This is rather a big price to pay, especially when all the inconveniences and other drawbacks are taken into consideration.

This gives a fair outline of the system as it is.

### The Remedy.

Those who wish to realise to the full the unpleasantness of the department from a hygienic point of view cannot do better than spend a couple of hours in the out-patient waiting-room of one of the large general hospitals, working under the conditions under which the doctors, nurses, and attendants have to work, or trying to put himself wholeheartedly in the patient's place. If such an investigator comes home after his day's experiences with any desire for dinner, he may be regarded as an exceptionally seasoned individual; if he escapes without encountering any of the dangers to health that lurk in the department, he will be lucky.

We have pointed out where the department fails; we have no need to point out the remedy. Let every manager, every committeeman, every friend of the institution make a point of visiting out-patients; let them study the hygiene of the place, its ventilation, its capacity, its comforts; let them see to it that some sort of system is introduced, an arrangement whereby batches of cases may be seen; above all, let them insist that the numbers of out-patients must be diminished in proportion to the ability of the staff to deal quickly and efficiently with them and the capacity of the department to contain them without necessitating the urgent visit of the sanitary inspector or the local medical officer of health. It is a disgrace to a modern hospital to allow a Black Hole of Calcutta to be on its premises under the guise of an out-patient department. It is a scandal that institutions which tolerate such Black Holes should have a large number of out-patients treated during the year to boast of.

